# 

**Published:** 1912-04-27

**Authors:** 


### Buildings Contemplated.

Ipswich.—The work of extending the nurses' home at the East Suffolk Hospital has been commenced, and the time for completion fixed at six months hence. The addition when completed and furnished will cost £2,500, and give sleeping accommodation for sixteen additional nurses.

Barnet.—The Barnet Hospital Committee have appointed Mr. Pill, of Messrs. White, Son and Pill, to advise them and carry out extensions to the isolation hospital; the approximate cost of the work is given as £3,000.

Balham.—Designs have been submitted in competition for the Weir Hospital to the trustees of the Weir Charity. The scheme of Mr. R. J. Thomson, of Wimbledon, has been selected.

Bishopstoke.—A tender of £4,880 has been accepted for the erection of an isolation hospital for the Eastleigh and Bishopstoke Urban Council.

### OUR BUREAU OF INFORMATION.

### The Brotherhood of Service.

The establishment of a Bureau of Information in The Hospital was the outcome of an often expressed want for some medium of communication between the public and the institutions which exist for their benefit. The common ignorance among ordinary people of the agencies at work for the cure and alleviation of their infirmities, whether mental or physical, has often proved a serious impediment to good work carried on without advertising. How often in lay papers and light literature, for instance, do we not see that "there ought to be a home" for this or that class of sufferers, who are amply provided for if the writers but knew it in homes which frequently lack patients. How often are persons discouraged from obtaining curative treatment which is intended for them by fears and doubts about the right kind of institution for their needs. The Bureau is intended for the service of those who need information, of those who need help, and of those who are qualified to give help and information. But the establishment of the Bureau and its proper development call for a closer link between those who give, those who receive, and those who provide the means of cure; between the Good Samaritan, his suffering neighbour, and the Innkeeper, for the part of the latter in the healing scheme is too often overlooked. This link we hope to see established in the Brotherhood of Service.

### What Kind of Members are Wanted.

If the Bureau is to be of service to the persons in want of institution treatment it follows that it must be in touch with social workers all over the country. Among those who get closest to the saddening knowledge of how much needs doing to bring relief to the suffering are the clergy and other religious ministers. We want them first. All qualified workers in the social field, members of settlements, and parochial helpers are eligible to join the Brotherhood. All fully trained nurses, health visitors, and almoners will be welcomed. Matrons and secretaries of hospitals will be specially valuable to the Brotherhood, on account of their opportunities of advising us in matters relating to institution vacancies and needs. In addition we want that section of the general public which aims at intelligent giving, and is on the watch to bring assistance to the poor, the suffering, and the friendless.

### What the Bureau can Offer.

First and foremost the Bureau offers the opportunity for making known the perplexities of those who are trying to help their neighbours. It can introduce the needs of sufferers, lacking in influence no less than in every other advantage, to a wide circle of sympathetic readers whose lives are spent in doing good. Many altogether hopeless cases considered in the light of such experience as a small parochial relief committee can bring to bear upon them are capable of curative treatment and may be restored to health and independence if proper co-operation can be secured between the Good Samaritan and the Inn. Again the Bureau, besides giving a hearing to properly authenticated tales of distress, can give expert advice and information to those who can pay, but not very heavily, for the required institution aid. The Bureau is making a special feature of collecting particulars of reputable homes willing to receive patients on very moderate terms. There are more of these than the public has any idea of, and we are taking considerable pains to compile a list of such as can be recommended by trustworthy persons. In addition the Bureau offers information on every subject of interest to readers who are taking part in the social movement of the day, and it will give particulars of how to obtain training and employment in different branches of philanthropic or institutional work.

### What the Brotherhood can Give.

The above advantages can only be secured to our readers if the Brotherhood of Service becomes a real interest in the lives of those who belong to it. It must become a chain which consists of clasped hands right round the world. Those who have information to give must give it freely and instantly. Those who can suggest an expedient must be prompt in bringing it to our notice. Those able to help must unite with us in spreading the good news to those who are in need. Nothing is so infectious as pity. Nothing is so cumulative in effect as the wish to help. From time to time the Bureau will look to members of the Brotherhood for help it can obtain in no other way. It must refer to them for information in their own locality; it must rely on their reports; it must look to them to suggest responsible persons to carry out investigations they may be unable to undertake themselves. In all parts of London, in every country town, in every country locality, in every part of the Colonies, the Bureau needs workers and will welcome help.

### NEEDS AND HELPERS.

### The Stolz Ear Treatment.

"W. G."—Can you tell me what is the Stolz ear treatment, which, I am told, is massage of the tympanum? It is not advertised, and doubts have been expressed whether it is quackery.

There is no recognised form of treatment known by this name, but it appears simply to be synonymous with the use of a well-known type of hearing instrument variously known as microphone, electrophone, etc. The apparatus associated with the name Stolz is of this type—a modified telephone, in fact. This particular machine is certainly widely advertised, and does aid the deaf, but just as certainly it does not cure them. It is conceivable that the continual use of such an apparatus may be claimed to constitute a "treatment," and it may also be *suggested* that such a "treatment" may "train" the impaired auditory nerves to do better things. This is as near a "Stolz treatment" as I can get after consulting an aural surgeon. Massage of the tympanum is another matter, and one of rather equivocal benefit too.

Home for Child.

"M. E. J.": See Rule 3. You must apply to have your home placed on our List. We cannot recommend unless we know something at first hand about your home, and our charge for this registration is 5s.

Fever Hospitals Affording no Proper Training.

"J. S. M.," Eastbourne.—Regret your letter did not reach us in time for reply last week. Your daughter's case is a very hard one, and we fear she is not the only victim to this kind of deception. Legal proceedings are not to be recommended, as they would be costly, vexatious, and very doubtfully successful. Communicate with the Hon. Secretary, the Fever Nurses' Association, Plaistow Fever Hospital, London, E. This association will supply names of fever hospitals giving really satisfactory training, and also maintains a list of nurses who are properly qualified in this branch of nursing, either before or after taking general training. We do not think the authorities of the hospital in question will court exposure by retaining your daughter's testimonials after a formal demand for them from yourself.

Course of Study in Radiography and Electricity.

"Yorke."—An excellent and very practical little booklet—"Electro-Medical Instruments and their Management"—is issued by Mr. Schall, of 75 New Cavendish Street, W., and you would probably be able to obtain private lessons from the same firm. Messrs. Davidson and Co., 29 Great Portland Street, undertake to give private lessons in the use of electrical apparatus. You should read a good text-book on physics and one or more of the following: "Medical Electricity," by Lewis Jones, M.D. (H. K. Lewis, 12s. 6d.); "Medical Electricity and Röntgen Rays," by Sinclair Tousey, M.D. (W. B. Saunders, 30s.); "A Manual of Practical Medical Electricity," by Dawson Turner, M.D. (Ballière, Tindall and Cox, 10s.). A good knowledge of photography must be obtained before you can become a successful radiographer. If possible you should obtain permission to visit the *x*-ray department of some hospital and there obtain an insight into the daily routine. You can learn many valuable practical details by watching an electrician fitting up an ordinary house installation and by studying the method of wiring such an installation. It should be borne in mind, however, that an unqualified person taking up electro-medical treatment is in a somewhat ambiguous position.

To Proprietors of Approved Homes.

The Editor of the Bureau is ready at all times to forward replies addressed c/o the Bureau to other correspondents from *members having Approved Homes entered on our List,* should they have reason to think that cases mentioned in our columns would be suitable for reception in their establishments.

It is impossible for the Editor, however, to put correspondents into communication with homes which have not previously been registered after inquiry into their *bona fides*, and no exception can be made to this rule.

### Rules for Correspondents.

Everyone who uses or wishes to help our Bureau of Information must be kind enough to observe the following rules:—

(1) Postal replies cannot be given. Exception will only be made in very special cases at the Editor's discretion.

(2) Applications referring to non-dependents where the need relates to business or employment must be accompanied by a postal order for 5s., when the requirement shall have publicity in these columns.

(3) All applicants for insertion in our list of Approved Homes must send a registration fee of 5s. The omission of this leads to delay and gives avoidable trouble.

(4) *All communications* for this department *must be accompanied by the coupon* to be found at the bottom of the third page of the cover on the inside, and should be addressed to the Editor of the Bureau of Information, c/o The Hospital, 29 Southampton Street, Strand, London, W.C.

### Appointments.

Mr. T. P. Gostling, senior house surgeon, has been re-elected to the staff of the Worcester General Infirmary.

Dr. William J. Gow, having resigned the appointment of physician to in-patients to Queen Charlotte's Lying-in Hospital, has been elected a consulting physician and vice-president of the hospital. For the vacancy thus created on the in-patient staff, Dr. Thomas G. Stevens, formerly physician to out-patients, has been selected, and Dr. Clifford White has been selected for the vacancy for a physician to out-patients.

The following appointments have been made at the Royal Free Hospital :—Male house surgeon, Mr. M. D. Mackenzie, M.B., B.S.; female house surgeon, Miss Rawlins, M.B., B.S.; female house physician, Miss Peake, M.B., B.S.; assistant anæsthetist, Miss Hood-Barrs, M.B., B.S.; senior obstetric assistant, Miss M. I. Waller, M.B., B.S.; senior clinical assistant to Gynæcological Department, Miss Mecredy, M.B., B.S.; junior clinical assistant to Gynæcological Department, Miss Cotton, M.B., B.S.; clinical assistant to Mr. Evans, Miss Watts, M.D.

Dr. Paton has been appointed senior house surgeon at the West Ham Hospital in the room of Dr. H. J. Windsor, resigned. Dr. Clark has been appointed junior house physician, and Mr. E. K. Martin, F.R.C.S., has accepted the appointment of examiner of nurses.

Mr. Albert Lucas, F.R.C.S., has been elected honorary surgeon to the General Hospital, Birmingham, for a term of fifteen years, in place of Sir Thomas Chavasse, whose resignation has been previously noted. Mr. Albert Lucas has been for nineteen years assistant surgeon, and was formerly resident surgical officer, and previously house surgeon at St. Bartholomew's Hospital, London, and resident medical officer at the Metropolitan Hospital. He is a Fellow of the Royal College of Surgeons of England (1891).

Dr. T. Arnold Johnston has been appointed hon. assistant physician to the Leicester Infirmary in the place of Dr. Robert Sevestre, promoted to the senior staff. Dr. Johnston graduated at Edinburgh with distinction and first-class honours, and was awarded high commendation for his M.D. thesis on "Original Research in the Pathology of Gastric Erosion." He has held senior resident posts in Edinburgh Royal Infirmary, Bradford Infirmary, and Leicester Infirmary, and has for the past eighteen months filled the post of pathologist to the latter institution.

The following appointments have been made at the Manchester Children's Hospital, Pendlebury: Mr. Glyn Evans, M.B., Ch.B. (Edin.), has been appointed junior resident medical officer; Miss Elsie Brown, M.B., Ch.B. (Manchester), has been appointed second assistant medical officer at the hospital's out-patients department, Gartside Street, Manchester; and Mr. E. A. T. Green, L.D.S. (Eng.), has been elected honorary dental surgeon in place of Mr. Baron J. Rodway, L.D.S., who has resigned after eleven years' service.

The current issue for April of *The Prescriber*, apart from its summary of therapeutic progress generally, devotes much of its space to the special consideration of diabetes. This, of course, increases the value of the journal, as it enables it to combine a wide field with a sufficient and distinctive specialism. It is understood that the June Number will deal specially with "Neurasthenia," when the developments in the treatment of that condition will be presented in a condensed form.

